# Intestinal Schistosomiasis and Giardiasis Co-Infection in Sub-Saharan Africa: Can a One Health Approach Improve Control of Each Waterborne Parasite Simultaneously?

**DOI:** 10.3390/tropicalmed5030137

**Published:** 2020-08-25

**Authors:** John Archer, Lisa O’Halloran, Hajri Al-Shehri, Shannan Summers, Tapan Bhattacharyya, Narcis B. Kabaterine, Aaron Atuhaire, Moses Adriko, Moses Arianaitwe, Martyn Stewart, E. James LaCourse, Bonnie L. Webster, Amaya L. Bustinduy, J. Russell Stothard

**Affiliations:** 1 Wolfson Wellcome Biomedical Laboratories, Department of Zoology, Natural History Museum, Cromwell Road, London SW7 5BD, UK; j.archer@nhm.ac.uk (J.A.); b.webster@nhm.ac.uk (B.L.W.); 2 Department of Tropical Disease Biology, Liverpool School of Tropical Medicine, Pembroke Place, Liverpool L3 5QA, UK; lisa.ohalloran@live.co.uk (L.O.); haj_3933@hotmail.com (H.A.-S.); martyn.stewart@lstmed.ac.uk (M.S.); james.lacourse@lstmed.ac.uk (E.J.L.); 3 Department of Tropical Infectious Diseases, Ministry of Health, Asir District, Abha 61411, Saudi Arabia; 4 Department of Clinical Research, London School of Hygiene and Tropical Medicine, Keppel Street, London WC1E 7HT, UK; shannan.summers1@student.lshtm.ac.uk (S.S.); tapan.bhattacharyya@lshtm.ac.uk (T.B.); amaya.bustinduy@lshtm.ac.uk (A.L.B.); 5 Vector Control Division, Ministry of Health, Kampala 759125, Uganda; vcdmoh@gmail.com (N.B.K.); aaronatuhaire@gmail.com (A.A.); adrikomoses@gmail.com (M.A.); vcdmoh@gmail.com (M.A.)

**Keywords:** One Health, *Schistosoma mansoni*, *Giardia duodenalis*, water, sanitation and hygiene (WASH), Uganda

## Abstract

Both intestinal schistosomiasis and giardiasis are co-endemic throughout many areas of sub-Saharan Africa, significantly impacting the health of millions of children in endemic areas. While giardiasis is not considered a neglected tropical disease (NTD), intestinal schistosomiasis is formally grouped under the NTD umbrella and receives significant advocacy and financial support for large-scale control. Although there are differences in the epidemiology between these two diseases, there are also key similarities that might be exploited within potential integrated control strategies permitting tandem interventions. In this review, we highlight these similarities and discuss opportunities for integrated control of giardiasis in low and middle-income countries where intestinal schistosomiasis is co-endemic. By applying new, advanced methods of disease surveillance, and by improving the provision of water, sanitation and hygiene (WASH) initiatives, (co)infection with intestinal schistosomiasis and/or giardiasis could not only be more effectively controlled but also better understood. In this light, we appraise the suitability of a One Health approach targeting both intestinal schistosomiasis and giardiasis, for if adopted more broadly, transmission of both diseases could be reduced to gain improvements in health and wellbeing.

## 1. Introduction

Throughout many tropical and sub-tropical low- and middle-income countries (LMICs) where provision of water, sanitation and hygiene (WASH) infrastructure is inadequate, communities of people are often found to be co-infected with multiple parasitic diseases acquired through ingestion of, or contact with, contaminated water and food [1,2]. Notably, intestinal schistosomiasis and giardiasis are both waterborne parasitic diseases highly prevalent and co-endemic in these regions. 

Intestinal schistosomiasis is a debilitating neglected tropical disease (NTD) caused by infection with parasitic blood flukes of the species *Schistosoma mansoni, S. japonicum, S. intercalatum, S. mekongi* and *S. guineensis* [3]. This disease is highly prevalent throughout many areas of sub-Saharan Africa and locally endemic in some areas of South America and the Caribbean, where the vast majority of cases are caused by infection with *S. mansoni.* Intestinal schistosomiasis compromises the general integrity of the small bowel via egg-induced perforations with associated local and systemic inflammation [3]. 

Giardiasis, another debilitating but underreported intestinal parasitic disease, is caused by infection with the single-celled eukaryotic diplomonad *Giardia duodenalis*, a flagellated protist [4]. While cosmopolitan in distribution, giardiasis prevalence is particularly high in low and middle-income countries [5,6]. Like intestinal schistosomiasis, giardiasis also compromises the general integrity of the digestive tract. It does this through a major disruption of the gut microbiota, causing a variety of debilitating pathologies such as dehydration and anemia [7,8]. Unlike intestinal schistosomiasis, however, giardiasis is not considered an NTD, although there have been previous discussions proposing its inclusion [9,10].

Whilst there are differences in the transmission biology and epidemiology between both intestinal schistosomiasis and giardiasis, there are also key similarities that might be exploited within potential integrated control strategies, allowing for control of both diseases in tandem. By highlighting these similarities, and by outlining opportunities for integrated control of giardiasis in areas where intestinal schistosomiasis is co-endemic, we aim to diminish the detrimental effects of (co)infection, thereby improving the health and wellbeing of those, particularly children, in endemic areas. To do this, an integrated ‘One Health’ approach is needed that requires a detailed knowledge of the transmission biology of each parasite, appropriate use of reliable point-of-care (POC) diagnostics, mitigation of environmental and zoonotic transmission through improved WASH infrastructure and effective use of anti-parasitic chemotherapies. Each of these should be carefully considered and applied simultaneously to improve public health outcomes [11,12,13,14]. 

## 2. Intestinal Schistosomiasis and Giardiasis: Pathology and Epidemiology

Intestinal schistosomiasis disproportionally afflicts school-aged children between the ages of six and fifteen years old, where pathology can be both acute and chronic [3]. As based on ‘classic’ age-infection profiles and measured using faecal egg counts, the intensity of infection typically begins to decline in late adolescence while morbidity associated with *S. mansoni,* such as multi-organ fibrosis, accumulates. This decline in egg-patent prevalence is due to a variety of factors such as partial-immunity to infection, notwithstanding extensive fibrosis of the bowel itself which can occlude egg exit sites, giving rise to granulomatous masses known as intestinal ‘bilharzomas’ [15,16].

Pathologies associated with intestinal schistosomiasis occur primarily as a result of the body’s response to the copious number of eggs produced by female adult worms inhabiting the mesenteric veins surrounding the intestines. Rather than being passed in stool (or occasionally in urine) to perpetuate the parasites lifecycle, a large proportion of eggs will instead become sequestered throughout the venous bloodstream of the intestinal and hepatoportal tracts. Many eggs will then enter into general venous circulation and subsequently become lodged in other major organs. Once eggs become trapped, for example in the intestinal wall and/or liver sinuses, a range of clinical systemic and organ-specific morbidity ensues, including acute abdominal pain, stunted growth, environmental enteropathy, presence of faecal occult blood and overt hepato/splenomegaly [17,18].

Human giardiasis is caused by infection with *G. duodenalis* (syn. *Giardia intestinalis, Giardia lamblia*). While *G. duodenalis* is the only human-infecting *Giardia* species, eight distinct evolutionary assemblages based on multi-locus genotyping, named A through H, are known to exist [19]. Of these eight, the vast majority of human infections are caused by assemblages A and B, although human infections with assemblages C, D, E and F do also occur, albeit rarely [19,20]. 

Unlike the distribution of *S. mansoni*, which is intrinsically linked to its *Biomphalaria* spp. intermediate freshwater snail hosts, the distribution of *G. duodenalis* is truly cosmopolitan. Giardiasis prevalence in humans is particularly high; however, in LMICs lacking access to clean, safe drinking water and associated WASH infrastructures, including many areas of sub-Saharan Africa and South America, where *S. mansoni* is also endemic [5,6]. A notable feature of giardiasis, in humans, can be asymptomatic infections, although acute and/or chronic and debilitating pathologies owing to infection are well described. These include diarrhoea, dehydration, malabsorption, tropical enteropathy, stunted growth, impaired cognitive development, anaemia and chronic fatigue [7,8]. The primary cause of these pathologies is a major disruption to the gut microbiota, a complex community of symbiotic microbes responsible for vitamin production, nutrient absorption and regulation of lipid metabolism, brought about through *G. duodenalis* invading, inhabiting and multiplying within the intestinal tract [21,22,23]. Importantly, severe morbidity is most often observed in certain high-risk groups including children, the elderly, those with physical disabilities and the immunocompromised [24,25].

### 2.1. Common Modes of Environmental Contamination

**Figure 1:**
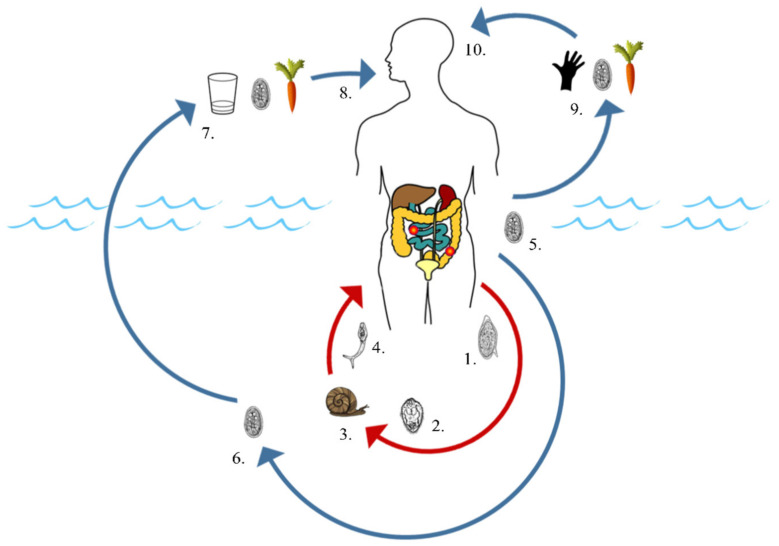
Transmission routes of *S. mansoni* (red, 1–4) and *G. duodenalis* (blue, 5–8 and 9–10). Both parasites are transmitted through faecal contamination of freshwater (*S. mansoni*: 1, *G. duodenalis*: 5). Infection with *S. mansoni* primarily occurs through cercarial penetration of the skin upon contact with contaminated water (4). Waterborne infection with *G. duodenalis* occurs through consumption of contaminated water or foods washed with contaminated water (7–8). Infection with *G. duodenalis* can also occur through consumption of contaminated foods or unwashed hands (9–10). Adapted from [26,27].

A major factor linking the transmission of both intestinal schistosomiasis and giardiasis is their transfaecal environmental contamination routes via the excretion of schistosome eggs (*S. mansoni*) or cysts (*G. duodenalis*) into a viable body of freshwater. Although waterborne transmission is not required for *G. duodenalis* to complete its life cycle, and while not all *S. mansoni* eggs or *G. duodenalis* cysts will successfully reach a viable freshwater habitat, in a disease-endemic setting, many environmental water bodies will undergo some extent of direct or indirect faecal contamination with both parasites (Figure 1) [3,4,26,27]. Indirect faecal contamination of freshwater can occur, for example, while bathing, through infected stools deposited on the banks of rivers and ponds being washed into these waters by heavy rains or floods, through overflowing pit latrines, and possibly through animals such as cattle walking through sites of defecation and transporting faecal matter to bodies of water on their hooves [28,29]. *S. mansoni* eggs can survive up to approximately eight days in the stool post-defecation and before reaching freshwater, whereas *G. duodenalis* cysts can survive up to eight weeks in the environment [4,30] (Table 1).

**Figure 2:**
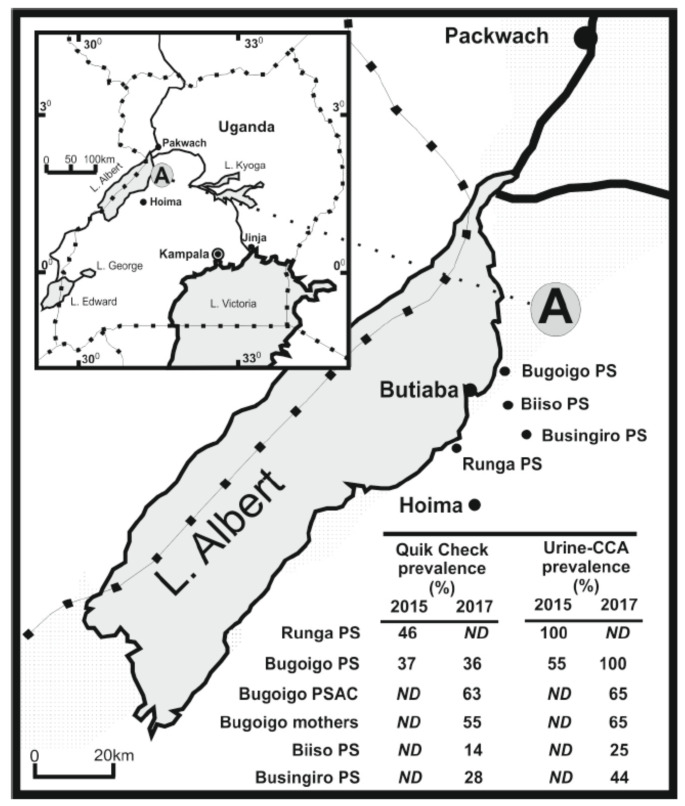
High prevalence of intestinal schistosomiasis (assessed using urine-CCA POC-RDT) and giardiasis co-infection (assessed using Quik Chek POC-RDT (TechLab, USA)) in school-aged children across multiple communities along the shoreline of Lake Albert, Uganda in 2015 and 2017 [8].

**Figure 3:**
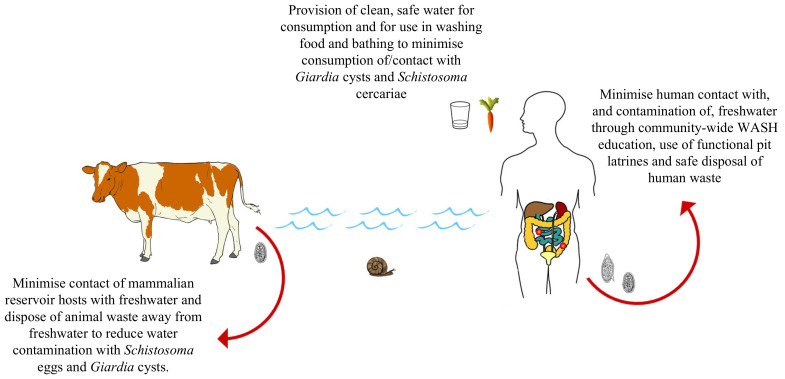
Examples of water, sanitation and hygiene (WASH) initiatives implemented to prevent the contamination of freshwater with *S. mansoni* eggs and *G. duodenalis* cysts, as well as to prevent contact with and consumption of contaminated water. Adapted from [26,27].

Once exposed to freshwater, *S. mansoni* eggs (Figure 1, (1)) will hatch to release free-swimming ciliated miracidia (Figure 1, (2)) that will then employ a range of morphological adaptations and host-seeking behaviours to locate and penetrate the soft tissues of its freshwater snail intermediate host, *Biomphalaria* spp. (Figure 1, (3)) [31,32,33]. Miracidia are ephemeral, living only a short period of time, typically less than six hours, before dying as their glycogen energy reserves are exhausted (Table 1). 

Miracidia that successfully invade a suitable intermediate snail host metamorphose into mother sporocysts, which, in turn, produce daughter sporocysts. These daughter sporocysts then differentiate upon sporogenesis, producing numerous cercariae that are shed from the snail approximately one month after initial invasion by the miracidium (Figure 1, (4)). Once established, cercarial production and shedding from *Biomphalaria* spp. snail hosts occur daily and typically continue over the remainder of the snails’ lives. Over the course of an infected snail’s life, tens of thousands of cercariae can be liberated [3,34]. 

Shed cercariae will then go on to infect humans and other mammalian definitive hosts, primarily through cutaneous penetration (Figure 1, (4)), although infection may also occur through penetration of the buccal cavity when consuming contaminated water [30]. Like miracidia, cercariae are ephemeral in freshwater as their glycogen energy reserves are quickly depleted, lasting no longer than three days (Table 1). In addition, survival of both miracidia and cercariae is highly dependent on favourable biotic and abiotic environmental conditions. Freshwater too high in salinity or too polluted, for example, can prevent the hatching of eggs into miracidia and can be lethal to both miracidia and cercariae [32,33,35,36].

*G. duodenalis* has a direct, faecal–oral life cycle that can be completed either through waterborne transmission when consuming water contaminated with cysts (or foods/utensils washed using contaminated water without sufficient soaps) (Figure 1, (5–8)), or through the consumption of contaminated foods or unwashed hands (Figure 1, (9–10)) [4,37]. Unlike *S. mansoni,* which will asexually reproduce within an intermediate host, and although *Giardia* cysts may survive and even accumulate within certain filter-feeding freshwater invertebrates (for example, oysters), *G. duodenalis* does not require an intermediate host for transmission [38]. Cysts passed in the stool are, however, extremely resilient and can remain viable within the stool or in freshwater for up to eight weeks after excretion (Table 1) [4,37,39]. 

Maintained transmission of intestinal schistosomiasis is therefore dependent on the continued contamination of freshwater and continued exposure to contaminated/infested water, whereas giardiasis transmission is heavily exacerbated by the continued contamination of freshwater and continued consumption of contaminated water. There are a variety of ways in which an individual can be exposed to and infected with both parasites, for example, when water is used for consumption, sanitation purposes, income generation from fishing or farming and/or recreation [40,41]. As such, transmission of both diseases is particularly high in impoverished areas lacking adequate WASH infrastructures, such as access to functional pit latrines and clean drinking water, as well as behavioural impediments in those unaware of how to avoid contamination and infection [42,43].

### 2.2. Transmission via Non-Human Hosts

The transmission of both intestinal schistosomiasis and giardiasis is also exacerbated by a range of non-human definitive hosts acting as either major or minor reservoirs of infection, although the precise extent to which these hosts contribute to human transmission is not fully understood (Table 2). Further to the significant health and economic impact of infection with African schistosomes and/or *Giardia* on, for example livestock, animal reservoirs of both parasites also pose challenges in controlling and reducing human transmission as each parasite follows similar routes of infection, contamination and, ultimately, environmental transmission via non-human hosts [5,44,45]. 

To reduce human transmission effectively, animal reservoirs and the degree to which they contribute to and maintain disease transmission must therefore be carefully considered when developing, implementing and monitoring any disease control strategies. Moreover, particular attention is needed on those animal hosts able to reintroduce parasites into viable bodies of freshwater following prior control or elimination campaigns [52]. Limiting contact of cattle with freshwater, for example, as well as limiting run-off from fields on which cattle manure has been spread and disposing of animal waste away from bodies of freshwater are known to reduce transmission of *Giardia,* as well as non-human infecting *Schistosoma* species [53,54]. Doing so, however, can be extremely challenging to implement and maintain through time. 

In light of recent findings, additional consideration should also be given to the potential emergence of schistosome hybrids and their impact on schistosomiasis transmission [55,56,57]. *Schistosoma mansoni*, for example, can form hybrids with rodent-infecting species *S. rodhani*, which have been observed along the shoreline of Lake Victoria, one of the Great East African lakes. However, *S. rodhaini* appears exclusive to rodents, together with the *S. mansoni-rodhaini* hybrids, but with many gaps in routine surveillance this appraisal may be incomplete [58,59]. Uniquely among trematodes, schistosomes are dioecious, and so adult worms form inter-species copulatory pairs which facilitate permissive introgression(s). In nature, pre- and post-zygotic reproductive isolating barriers, such as host specificity, anatomical site of infection, geographical distribution, mating preference, worm competition and snail incompatibility, are thought to prevent prolific inter-species admixture. Recently, however, and owing to advanced methods of molecular analysis on schistosome larval stages from snail-intermediate and mammalian-definitive hosts, surprising inter-species hybrid forms are now being identified in several endemic African countries [58]. Such hybrids, resulting from interactions between human- and animal-infecting species, not only raise concerns about zoonotic transmission, but also the expanded host ranges and increased transmission potential acquired through heritable traits [59]. 

Changes to natural landscapes can readily lead to the formation of new freshwater bodies, snail habitats and multi-host transmission sites, breaking down the ecological barriers between species and leading to further inter-species interactions. Although the full impact that these hybridization events may have on human disease epidemiology and disease pathology is currently unknown, hybridization certainly suggests that future schistosomiasis control may warrant an expanded One Health approach with more tailored interventions specific to local settings and schistosome epidemiology [56,58,60].

## 3. Intestinal Schistosomiasis and Giardiasis: Surveillance and Control

Highlighting these key similarities in disease transmission, biology and epidemiology between *S. mansoni* and *Giardia* presents clear opportunities for integrated surveillance and control of both diseases, particularly with regard to disease diagnosis, surveillance and control.

### 3.1. Diagnosis: Parasitological, Immunological and Molecular Methods

Owing to its low cost, portability and high specificity, light microscopy for the detection of *S. mansoni* eggs in faecal samples is widely used during disease surveillance programmes to detect infection with *S. mansoni* in sub-Saharan Africa [61]. Using microscopy, routine parasitological surveillance to assess endemicity and prevalence of intestinal schistosomiasis, as well as other intestinal helminth infections, in a community is typically carried out via the Kato-Katz technique using faecal samples provided by school-aged children [62]. While inexpensive and portable, the sensitivity of Kato-Katz is, however, severely reduced in low-intensity infections, hampering its use in areas of low disease endemicity or in areas having undergone successful disease control intervention(s) [63,64,65].

Giardiasis cannot be reliably detected using the Kato-Katz method, and so alternative methods, such as formalin/ether concentration techniques, flotation techniques or immunofluorescent antibody microscopy, are needed [11,66,67,68]. Unfortunately, however, none of these techniques are straightforward or inexpensive to carry out under rural field conditions, particularly as it has been reported that formalin/ether concentration techniques have a higher sensitivity in detecting infection with *S. mansoni* than the more field-deployable Kato-Katz technique [69,70]. Moreover, these techniques can also often be insufficiently sensitive to reliably detect giardiasis infection [68].

For these reasons, a variety of immunological and molecular diagnostic assays with improved sensitivity in detecting both *S. mansoni* and *Giardia* infection using non-invasive urine and faecal samples have been developed (Table 3).

Though highly sensitive, immunoassays such as the enzyme-linked immunosorbent assay (ELISA) and molecular assays such as PCR/qPCR require specialist laboratory infrastructure seldom available in disease-endemic areas, preventing their use at POC [84,85]. As such, several rapid and field-deployable RDTs have also been developed to detect trace levels of parasite-derived antigens and parasite-derived DNA in urine and faecal samples. Some examples include straightforward lateral-flow dipsticks to detect *S. mansoni* circulating cathodic antigen (CCA) in urine samples and *G. duodenalis* (with or without *Cryptosporidium* spp.) cyst antigen in faecal samples, as well as loop-mediated isothermal amplification (LAMP) and recombinase polymerase amplification (RPA) assays to detect species-specific *Schistosoma-* and *Giardia*-derived DNA in urine and faecal samples [11,64,85].

While POC-RDTs have many advantages over light-microscopy, microscopy remains less financially expensive to carry out and so it is the favoured method of diagnosis during routine monitoring and control programmes with only limited financial resources available [68,75,78]. In addition, and though promising, assays such as LAMP and RPA to detect species-specific parasite DNA currently require further assessment and validation before their upscaled and routine use in such control programmes [81,85]. Nevertheless, continued development, assessment and validation of POC-RDTs is widely advocated as affordable and sensitive point-of-care diagnostics, capable of detecting low levels of infection within individuals able to maintain disease transmission, are sorely needed [86]. Given these challenges in reliably detecting infection using human samples, particularly in low-endemicity settings, alternative methods of detecting and monitoring disease transmission within endemic foci, such as parasite host surveillance and use of environmental DNA (eDNA), have also been explored.

### 3.2. Exploring the One Health InterFace with Increased Host Surveillance

Intermediate hosts and definitive reservoir hosts, such as *Biomphalaria* freshwater snails (*S. mansoni*) and rodents or cattle (*S. mansoni* and *G. duodenalis*, respectively), offer an alternative means of detecting and monitoring disease transmission in areas where detecting transmission through human diagnosis may be unreliable [87]. Collecting freshwater snails capable of transmitting schistosomes and carrying out shedding analyses to assess cercarial emergence, for example, may help identify active transmission sites [87,88]. This approach, however, can also be unreliable, as very few snails are typically found to be shedding cercariae [89]. 

For this reason, highly sensitive molecular xenomonitoring approaches to detect *Schistosoma* DNA in snail host tissues have also been developed and assessed [90]. Using PCR to detect *Schistosoma* DNA within snail hosts, for example, can identify prepatent infections and is not affected by diurnal fluctuations in cercarial shedding in the same way that cercarial shedding is; allowing a more reliable assessment of schistosome presence in a given locality than shedding analyses can allow. An added advantage of molecular xenomonitoring by use of PCR is the ability to genotype parasite and snail DNA, providing valuable opportunities to better understand disease transmission and molecular epidemiology, such as more reliable species identification of human-infecting cercariae and snail intermediate hosts than can be achieved using morphological analysis and the detection of *Schistosoma* hybridisation events [55]. In addition, collecting and screening freshwater snail hosts for the presence of parasite DNA can be more straightforward and more lucrative than collecting and screening human faecal samples for parasite DNA [90]. Currently, however, mass collection and molecular screening of freshwater snail hosts using PCR remains logistically, technically and financially demanding, and so development of a high-throughput methodology, possibly incorporating use of rapid and POC DNA amplification technologies such as LAMP or RPA, or pooling of snail samples, should also be further explored and assessed [91,92].

Similarly, molecular detection of *S. mansoni* and *G. duodenalis* DNA in DNA extracted from faeces collected from definitive reservoir hosts capable of perpetuating transmission, such as rodents (*S. mansoni*) and cattle (*Giardia*), has also been used to monitor disease transmission with success [49,58]. An added benefit of collecting and analysing faecal samples in this way is that this method also provides an opportunity to assess and better understand wild-type *Schistosoma* hybridisation events and zoonotic transmission of human-infecting *G. duodenalis* through genotyping [55,93,94]. Again, however, this approach too requires significant financial and technological resources. As such, it is unlikely to be widely integrated into control programmes undertaken in low-resource areas such as rural regions of sub-Saharan Africa without further development and use of field-deployable DNA amplification technologies.

### 3.3. Detecting Parasitic Contamination through Water Sampling and by Environmental DNA (eDNA) Analysis

Extensive screening of environmental water samples for contamination with *Giardia* cysts using the United States Environmental Protection Agency method 1623 (US-EPA method 1623) has been carried out in many areas of the world, such as the USA [95]. Through collection and filtration of water samples, protozoan cysts can be detected and quantified, allowing viable transmission sites to be identified [96]. Although straightforward to carry out, this method does not differentiate between morphologically identical *Giardia* assemblages A-H and so identification of human-infecting *Giardia* transmission sites, specifically, is not possible. As such, revised methods of detecting and monitoring assemblage-specific *Giardia*-contaminated water sources, such as through detection and genotypic analysis of parasite-derived eDNA, have been developed. 

Assessing and monitoring disease transmission within a given focus through the detection of parasite-derived eDNA rather than, or in conjunction with, using human bodily samples, has been explored with respect to a range of waterborne pathogens, including both schistosomiasis and giardiasis [97,98,99]. Dependence of both parasites on freshwater provides an ideal target for sample collection and assessment using PCR/qPCR, LAMP or RPA assays [83,100,101]. In addition, collection of water samples to detect eDNA derived from *Schistosoma* freshwater snail hosts to identify and monitor the presence of snail species capable of transmitting infection within a given waterbody has also been assessed [102,103].

Again, though promising, the upscaled and routine use of molecular assays to detect parasite- and/or parasite host-derived eDNA remains beyond the financial reach of most LMIC control programmes and too requires further methodological development, assessment and validation. Nevertheless, continued development of this approach to better understand the potential of eDNA as an effective monitoring tool and to reduce associated financial costs has been encouraged [75]. In particular, and like molecular xenomonitoring approaches, the monitoring of eDNA to identify disease transmission may prove extremely useful in areas of low disease endemicity where identifying infection in individual patients may be challenging.

### 3.4. A Case Example of Co-Infection and Morbidity Surveillance in Uganda

Given these key similarities in disease transmission biology and co-endemicity of both intestinal schistosomiasis and giardiasis throughout much of sub-Saharan Africa, co-infection with both parasites is likely commonplace, yet only little formal attention has been given towards co-surveillance of both diseases. This is despite each parasite potentially influencing reciprocal infection susceptibilities and disease-associated pathologies, before and after anti-parasitic treatment(s). As an example, it is possible that *S. mansoni* egg-induced perforations to the bowel with associated mucosal bleeding, inflammation and bacterial translocation may influence an individual’s susceptibility to chronic *Giardia* infection. The extent to which this occurs, however, is currently unknown.

This lack of attention on co-infection and co-surveillance may be, in part, due to an unfortunate division within parasitology that often siloes macro-parasite (helminth) and micro-parasite (protist) research. Though sparse, recent epidemiological studies are now beginning to shed more detailed light on the prevalence of co-infection of intestinal schistosomiasis and giardiasis, with detection of associated morbidities, throughout rural areas of sub-Saharan Africa [11,104,105]. A suitable example arises from two recent studies assessing co-infection in school-aged children along the shoreline of Lake Albert, Uganda, which, despite ongoing preventive chemotherapy for intestinal schistosomiasis, can still be considered hyper-endemic for *S. mansoni* today (Figure 2) [88]. Here, initial infection with *S. mansoni* occurs very soon after birth, with all ages vulnerable to infection and chronic disease [106]. 

Beginning in 2015, Al-Shehri et al. [8] conducted a novel attempt to integrate surveillance for intestinal schistosomiasis, giardiasis and malaria using available POC rapid diagnostic tests (RDTs) combined with later real-time qPCR analysis of stool and finger-prick collected blood with parasite-specific TaqMan DNA^®^ probes. This was the first attempt to quantify giardiasis with the POC Quik Chek RDT (TechLab, USA), finding 42% of children attending Runga and Bugoigo primary schools to be positive (Figure 2). Upon qPCR analysis of ethanol preserved stool using an 18S rDNA *Giardia*-specific TaqMan^®^ probe, up to 87.0% of children were found excreting *Giardia* DNA. Notably, the prevalence of heavy infection by real-time PCR (Ct ≤ 19) was 19.5% and strongly associated with Quik-Chek RDTs, as well as postively correlated with increasing intensities of egg-patent schistosomiasis and host anaemia [11].

*Giardia* species assemblages present were also later identified and characterised with specific triose phosphate isomerase (TPI) Taqman^®^ probes and by sequence characterisation of the *β*-giardin gene [107]. While less sensitive than the 18S rDNA assay, general prevalence by TPI probes was 52%, with prevalence by taxon assemblage of 8% (assemblage A), 36% (assemblage B) and 8% co-infection (A and B assemblages), and while assemblage B was dominant across the sample, proportions of assemblages A and B, and co-infections thereof, varied by school and by age of child. Mixed infections were particularly common at Runga school and in children aged 6 and under. Most importantly, infection with assemblage B was associated with underweight children. The presence of each assemblage was also confirmed by sequence analysis of the *β*-giardin gene finding sub-assemblage AII and further genetic diversity within assemblage B; also of note was the absence cryptosporidiosis, another pertinent water-borne disease, concurrently detectable by the same Quik Chek RDT.

To assess any changes through time, a repeat epidemiological survey was undertaken in 2017 which included reinspection of Bugoigo school and expanded point-of-care testing with Quik Chek (Figure 2). The prevalence of giardiasis at Bugoigo primary school was shown to be identical with a third of children examined positive by Quik Chek, with even higher local prevalence in pre-school-age children (63%) and their mothers (55%), good evidence for pervasive nature of giardiasis across all ages. Away from the lake at Biiso and Busingiro, the prevalence of giardiasis and intestinal schistosomiasis declined, suggesting that the risk of infection is perhaps higher on the lake shoreline. This study also attempted to evaluate a new POC-RPA RDT onsite, as well as a pilot assessment of giardiasis in local livestock and companion animals [81]. Ultimately, the RPA assay did not perform as well as expected, being in need for further optimisation of stool DNA extraction protocols. 

Further to human-surveillance, screening for *S. mansoni* transmission has also taken place along the same shoreline by collection and cercarial shedding of *Biomphalaria* freshwater snail hosts with success [88,108]. Future surveillance should also be carried out through molecular xenomonitoring of collected snails (and so omitting the need for shedding analyses), collection and screening of non-human definitive hosts to identify *S. mansoni* and *G. duodenalis* DNA and through collection and screening of surface water samples to identify *S. mansoni* and *G. duodenalis* eDNA.

### 3.5. Access to Treatment and Large-Scale Campaigns

In areas where schistosomiasis transmission is identified, preventive chemotherapy through repeated mass drug administration (MDA) of the donated anthelmintic drug praziquantel (40 mg/kg body weight) is the principal strategy for disease control [109]. Because the highest burden of infection is typically seen in children and young adolescents, MDA is customarily carried out in schools, but aims to limit overall transmission within a community through a reduced human reservoir of infection while also reducing overall disease morbidity [110]. Though praziquantel’s mechanism of action is not currently fully understood, significant reductions in disease prevalence and morbidity have been seen globally since MDA programmes began in 2001 [111,112]. Reinfection of schistosomiasis following treatment is, however, commonplace owing to communities’ reliance on freshwater, and so MDA must be repeated annually or biannually, depending on disease prevalence, to achieve a sustained impact.

Severe adverse effects are seen only very rarely when distributing praziquantel, making it well suited for mass distribution. Praziquantel, however, typically does not achieve 100% infection clearance primarily because dosing is usually based only on height and so does not account for differences in body mass. As a result, treatment success varies between individuals meaning many are still able to continue maintaining transmission [113]. In addition, and while local school systems provide a viable means of mass-distributing praziquantel, important human reservoirs of infection, including pre-school-aged children and adults, typically remain untreated [106,114]. 

The need for repeated annual or biannual distribution of MDA in this way has also raised regular concerns about the development of praziquantel resistance in schistosomes; particularly as there is currently no known efficacious alternative treatment to replace praziquantel if *Schistosoma* populations were to become more drug-tolerant or resistant [115,116]. A significant reduction in praziquantel efficacy, identified by a decreased reduction in *Schistosoma* egg output from infected individuals pre- and post-praziquantel treatment, has already been reported in *S. mansoni* populations in many communities across sub-Saharan Africa that have undergone repeated rounds of MDA [113]. This reduced efficacy may be a direct result of selection pressure placed on schistosomes during repeated and prolonged MDA campaigns, highlighting an urgent need to consider alternative methods of disease control outside of MDA.

A variety of drugs can be used to treat giardiasis [117,118]. Of these, metronidazole is the most predominantly used and most thoroughly studied owing to its straightforward oral administration and relatively low price. Like with schistosomiasis, reinfection with giardiasis is also commonplace; however, repeated mass drug administration to alleviate giardiasis transmission is not seen as a feasible strategy because the drug is not currently involved in any donation scheme, severe adverse effects of treatment are often seen, and metronidazole has only limited efficacy in clearing infection [117]. As an example, it has been reported that just one course of treatment has only an approximately 60% clearance rate, and so repeated treatment is needed to significantly clear infection [119]. Repeated treatment, however, not only significantly increases the likelihood of severe adverse events but is difficult to carry out during MDA campaigns [5]. In addition, the potential emergence of giardiasis resistance to treatment with metronidazole has also recently been reported, and while alternative and more efficacious chemotherapies, such as tinidazole exist, these can also cause adverse events [117,118,119]. Albendazole, a broad-spectrum and efficacious anthelmintic treatment used in MDA campaigns to reduce transmission of soil-transmitted helminth and some filarial nematode infections, can also be used to treat giardiasis [117,120,121]. To significantly reduce *Giardia* infection, however, a minimum dosage of 200–800 mg/day albendazole is needed for at least three concurrent days which, again, is difficult to carry out in the context of MDA campaigns and, by having limited donated stocks, also diminishes albendazole availability for anti-helminth control programmes [117,118,122].

Though treatment of schistosomiasis and giardiasis using praziquantel and metronidazole are important components of disease control, aligning treatment of both diseases in tandem during control campaigns may be challenging, owing to differences in drug type, dosages and treatment courses. In addition, as neither drug can safely and reliably achieve a 100% clearance rate using just one dosage, it is now widely accepted that alternative methods of control to reduce transmission and overall prevalence must be implemented alongside treatment campaigns if disease elimination targets are to be met. One such example is the implementation of WASH initiatives in communities where both diseases are endemic. The extent to which WASH provision, when used in conjunction with MDA, can successfully reduce schistosomiasis transmission is now beginning to be understood, and although only minimal data has been reported on the impact of WASH provision on giardiasis transmission in sub-Saharan Africa, it is widely assumed that improved WASH infrastructure would help significantly reduce giardiasis transmission [1,123,124].

### 3.6. Water, Sanitation and Hygeine (WASH)

WASH provision and infrastructure is extremely inadequate throughout many areas of rural sub-Saharan Africa [125]. In 2012, the World Health Assembly (WHA) formally advocated for the integration of WASH provision and education initiatives into amenable NTD control and elimination programmes; subsequently publishing guidance on ways in which these can be integrated [126,127]. Since, much attention has been given towards how WASH initiatives can be tailored for use, specifically, in schistosomiasis control programmes and the impact such initiatives have had when used in tandem with routine strategies such as preventive chemotherapy [2,30,128,129].

WASH initiatives relevant to schistosomiasis control, such as the adequate provision of safe drinking water, fully functional and properly maintained pit latrines and improved community hygiene education, effectively reduce disease transmission by minimising the direct and indirect contamination of freshwater by infected individuals and animals, by reducing contact with/consumption of infectious waters by human and non-human hosts and by helping communities better understand human and non-human disease transmission [30,130] (Figure 3).

Like intestinal schistosomiasis, giardiasis is widely prevalent throughout many rural and impoverished regions of sub-Saharan Africa and is intrinsically linked to contact with contaminated and unsafe water in areas lacking adequate water, sanitation and hygiene (WASH) infrastructure [4,41]. Giardiasis, however, receives only relatively little attention with regard to disease control, surveillance and elimination throughout sub-Saharan Africa. 

As an example, despite numerous clear advantages of implementing WASH initiatives on reducing schistosomiasis transmission and despite these key similarities between schistosomiasis and giardiasis with regard to disease transmission biology, surprisingly little attention has been given to the impact of improved WASH provision and education on giardiasis transmission in sub-Saharan Africa [43]. This oversight presents not only a missed opportunity with regard to better understanding, and reducing, giardiasis transmission and its associated pathological impact on some of the world’s most disadvantaged communities, but also presents the question: why is giardiasis ignored in intestinal schistosomiasis monitoring and control programmes?

## 4. Intestinal Schistosomiasis and Giardiasis: Towards a One Health Approach

Research funding opportunities for NTDs are limited when compared to those for other diseases such as human immunodeficiency virus (HIV), malaria or tuberculosis [131]. One way in which the impact of NTD control programmes can be significantly increased, however, is by appropriate integration with other disease surveillance, control, research and policy efforts. Successful examples of this integrated One Health approach can be seen when integrating lymphatic filariasis surveillance and elimination efforts into malaria elimination programmes, as well as by integrating soil-transmitted helminth and schistosomiasis control and elimination efforts [128,132,133,134,135]. 

In keeping with this integrated One Health approach, here, we propose a variety of ways in which the transmission of, and pathologies associated with, co-infection of intestinal schistosomiasis and giardiasis can be better understood, monitored and reduced via the integration of giardiasis control efforts into existing schistosomiasis control programmes. These include:

- Integrating screening of giardiasis endemicity and infection prevalence into existing schistosomiasis control programmes by using stool samples used for diagnosing infection with *S. mansoni*, and other intestinal parasites, to also record and report levels of *Giardia* infection in school-aged children. This can be conducted with POC-RDTs such as the Quik Chek immunoassay or using PCR/qPCR. In addition, the continued development, assessment and application of sensitive and straightforward POC-RDTs able to detect low-levels of infection in asymptomatic individuals capable of maintaining transmission of both parasites, such as the RPA, is encouraged.
- Further development and application of sensitive molecular assays to detect trace levels of species/assemblage-specific parasite DNA within freshwater snail intermediate hosts of human-infecting *Schistosoma*, and in faecal samples from non-human animal definitive hosts of both diseases. Further development and application of sensitive molecular assays to detect trace levels of species-/assemblage-specific parasite DNA from human-infecting *Schistosoma* cercariae and *Giardia* cysts in water samples easily collected from viable transmission sites is also encouraged.
- The upscaled provision of water, sanitation and hygiene (WASH) infrastructure and education initiatives to communities afflicted by both schistosomiasis and giardiasis to reduce environmental contamination events and to reduce contact with/consumption of contaminated water, simultaneously reducing transmission of both diseases.
- Monitoring *Giardia* disease prevalence and associated morbidities in tandem with schistosomiasis surveillance in school-aged children following any control programme intervention to better understand how giardiasis transmission and related pathologies can be reduced.
- An increased focus on understanding how the transmission of intestinal schistosomiasis and giardiasis, as well as immune responses and morbidities related to both diseases, interact and are potentially exacerbated by co-infection.

## 5. Conclusions

Here, we have highlighted various potential opportunities to improve the health and wellbeing of individuals in low- and middle-income countries where intestinal schistosomiasis and giardiasis are co-endemic by exploiting key similarities between both diseases with regard to disease transmission biology, epidemiology, surveillance and control. In addition, future steps needed to develop and implement an integrated, One Health approach for intestinal schistosomiasis and giardiasis co-infection surveillance, control and elimination strategies, are also outlined. In adopting this One Health approach, and by integrating giardiasis surveillance, control and elimination efforts into existing schistosomiasis elimination programmes, not only can the debilitating pathological impacts of intestinal schistosomiasis/giardiasis co-infection be better understood, but a reduction in co-infection and concurrent reduction in disease-related morbidities experienced by the world’s most disadvantaged communities can also be achieved.

## Figures and Tables

**Table 1:** Survival and viability of *S. mansoni* eggs and *G. duodenalis* cysts in the environment post-defecation.

Species	Life-Stage	Survival Post-Defecation in the Stool or in Freshwater	Reference(s)
*S. mansoni*	Eggs	~8 days in stool prior to reaching freshwater	[30]
	Miracidia	<6 h in freshwater	[31,32,33]
	Cercariae	~1–3 days in freshwater	[32,33,35,36]
*G. duodenalis* (Assemblages A and B)	Cysts	Up to eight weeks in stool or in freshwater	[4,37,39]

**Table 2:** Primary reservoir hosts of *S. mansoni* and *G. duodenalis* (assemblages A-H). ‘+’ denotes known primary reservoir host; ‘-‘ denotes no known primary reservoir host.

Species	Humans	Non-Human Primates	Ruminants	Rodents	Other Mammals	Fish	References
*S. mansoni*	+	+	-	+	-	-	[46,47]
*G. duodenalis* (assemblage A)	+	+	+	+	+	+	[20,48,49,50,51]
*G. duodenalis* (assemblage B)	+	+	+	+	+	+	[20,48,49,50]
*G. duodenalis* (assemblage C)	- *	-	+	-	+	-	[19,20]
*G. duodenalis* (assemblage D)	- *	-	+	-	+	-	[19,20]
*G. duodenalis* (assemblage E)	- *	-	+	-	+	-	[19,20]
*G. duodenalis* (assemblage F)	- *	-	-	-	+	-	[19,20]
*G. duodenalis* (assemblage G)	-	-	-	+	-	-	[19,20]
*G. duodenalis* (assemblage H)	-	-	-	-	+	-	[19,20]

* Human infection possible but rarely observed [20].

**Table 3:** Overview of primary diagnostic assays to detect infection with *Schistosoma mansoni* and *Giardia duodenalis*.

Species	Direct Diagnosis	Antigen Detection	Molecular Diagnosis
*S. mansoni*	Identification of ova in concentrated faecal smear via Kato-Katz technique [62,71]	Detection of circulating cathodic antigen (CCA) or circulating anodic antigen (CAA) in urine samples using ELISA or lateral-flow test strips [72,73]	Detection and amplification of species-specific DNA in faecal samples using PCR/qPCR [74], (LAMP and RPA assays have also been developed [71,75,76,77]).
*G. duodenalis*	Identification of cysts in concentrated faecal smear via formalin/ether concentration techniques, flotation techniques or immunofluorescent antibody microscopy [68]	Detection of species-specific antigens in faecal samples using ELISA or lateral-flow test strips [11,78,79,80]	Detection and amplification of species-specific DNA in faecal samples using PCR/qPCR [78], (LAMP and RPA assays have also been developed [81,82,83]).

## Abbreviations

eDNA	environmental DNA
ELISA	enzyme-linked immunosorbent assay
FGS	female genital schistosomiasis
LAMP	loop-mediated isothermal reaction
LMIC	lower-middle-income countries
MDA	mass drug administration
NTD	neglected tropical disease
PCR	polymerase chain reaction
POC	point-of-care
qPCR	quantitative polymerase chain reaction
RDT	rapid diagnostic test
RPA	recombinase polymerase amplification
Th1	T-helper 1
Th2	T-helper 2
WASH	water, sanitation and hygiene
WHA	world health assembly
